# Challenges and opportunities associated with cervical cancer screening programs in a low income, high HIV prevalence context

**DOI:** 10.1186/s12905-021-01211-w

**Published:** 2021-02-18

**Authors:** Adebola Adedimeji, Rogers Ajeh, Amanda Pierz, Relindis Nkeng, Jackson Jr. Ndenkeh, Norbert Fuhngwa, Denis Nsame, Miriam Nji, Anastase Dzudie, Kathryn M. Anastos, Philip E. Castle

**Affiliations:** 1 Department of Epidemiology and Population Health, Albert Einstein College of Medicine, 1300 Morris Park Avenue, Bronx, NY 10461 USA; 2 Clinical Research Education, Networking and Consultancy, Yaoundé, Cameroon; 3 Center for International Health, Ludwig Maximillian University of Munich, Munich, Germany; 4 Limbe Regional Hospital, Limbe, Southwest Region Cameroon; 5 Rollins School of Public Health, Emory University, Atlanta, GA USA

**Keywords:** Cervical cancer screening, Human immunodeficiency virus (HIV), Cameroon, Low-income, Social determinants

## Abstract

**Background:**

Cervical cancer is a leading cause of death among Cameroon women. The burden of cervical cancer is in part traceable to the inadequate understanding of socio-contextual determinants of access to screening and prevention opportunities. We explored multilevel individual, community and structural factors that facilitate or inhibit cervical cancer prevention in women at risk in a low-income, high HIV prevalence context.

**Methods:**

We utilized an exploratory qualitative approach to obtain data through focus group discussions and in-depth interviews from May to August, 2018. A two-stage purposive sampling strategy was used to select 80 women and 20 men who participated in 8 focus group discussions and 8 in-depth interviews. The socio-ecological model guided data analyses to identify micro-, meso-, and macro-level determinants of cervical cancer screening.

**Results:**

Micro-level factors including lack of awareness and knowledge about cervical cancer, lack of access to information, excessive cost of cervical cancer screening, low risk perceptions, and poor health seeking behaviors were major barriers for women seeking cervical cancer screening. Meso-level factors, such as social networks, socio-cultural norms, perceptions of the role of men and HIV-related stigma when screening is integrated into HIV care, also engender negative attitudes and behaviors. Macro-level barriers to cervical cancer screening included poorly equipped health facilities and a lack of national cancer prevention policies and programs.

**Conclusion:**

In the context of the call for elimination of cervical cancer as a public health problem, our findings highlight challenges and opportunities that should be considered when implementing interventions to increase uptake of cervical cancer screening in low-middle income settings.

## Background

Invasive cervical cancer (ICC) is the 4th most common cancer, and a leading cause of death among women globally [1]. In 2018, more than 570,000 cases were newly diagnosed, representing 6.6% of all cancers in women worldwide [2]. Cervical cancer incidence and mortality vary widely by region, but some estimates suggest that more than 90% of cervical cancer deaths occurred among women in low and middle-income countries (LMICs) [2]. While high-income countries (HICs) have been able to limit cervical cancer incidence and mortality among women, those in LMICs continue to bear a disproportionate burden because they lack resources for prevention, early detection, and treatment.

The current rate of morbidity and mortality from cervical cancer among women in LMICs is mostly preventable through comprehensive cervical cancer prevention programs that include primary prevention through human papillomavirus (HPV) vaccination and secondary prevention through the effective treatment of cervical cancer precursors [2]. In HICs for example, the use of cervical cytology (Pap smear) and HPV testing to detect high-grade cervical intraepithelial neoplasia (CIN) has been routinely used to prevent ICC in women at risk. However, LMICs often have limited and inadequate health infrastructure, a lack of trained personnel, and exorbitant cost to patients that limit the feasibility and effectiveness of Pap smear tests in screening age-eligible women.

To address this problem, the World Health Organization recommended several cost-saving options that are equally effective at preventing or detecting cervical cancer in women living in LMICs. These include vaccination to protect from common types of HPV that are linked to cancer, and high-risk HPV (hrHPV) DNA testing and/or visual inspection with acetic acid (VIA) with same-day ablative treatment in mid-adult women who will have limited benefit from HPV vaccination [3, 4]. Despite these recommendations, many countries in sub-Saharan Africa (SSA) do not have population-based cervical cancer screening programs [5]. Women still face considerable challenges in accessing current cervical cancer screening programs. These include weak health systems, inadequate funding, and personnel to implement routine screening programs, exorbitant costs associated with screening, low level of awareness and education about existing programs, and late presentation and diagnosis [6]. To overcome these challenges, many countries are exploring strategies for effective cervical cancer screening and treatment programs, for example, integrating cervical cancer screening within HIV/AIDS programs to increase access for women at greatest risk, ensure sustainability and lower cost compared with stand-alone programs [7].

Cameroon, a culturally and ecologically diverse country located in sub-Saharan Africa, has an estimated population of over 25 million as of 2018, and women make up nearly half of the country’s population [8]. In 2018, 2,356 new cases of cervical cancer were diagnosed in women, which represented 14% of all cancers diagnosed in the country, and 25% of cancers diagnosed in women [9]. Despite this, the uptake of cervical cancer screening among women in Cameroon is less than 20% [10].

Although screening remains an effective strategy for secondary prevention of cervical cancer, many women in Cameroon encounter considerable barriers when accessing screening services. These included, for example, socioecological barriers associated with interpersonal, social, community and structural factors [11]. Other factors that limit women’s access to screening included inadequate information and access to existing screening options, the prohibitive cost of accessing existing services, poor health-seeking behaviors, stigma, poorly equipped health infrastructure, and other socio-cultural factors [10]. The success of cervical cancer prevention in Cameroon will depend on identifying effective strategies to eliminate these contextual challenges.

Multi-level models that transcend any single level of influence within the social ecology of health behavior, including individual/interpersonal (e.g. level of awareness and knowledge, behaviors related to accessing resources to promote health and wellbeing, perceptions and interpretations of disease), socio-environmental (e.g. family and social networks, community norms, stigma) and macro-structural factors (e.g. population-based programs, health infrastructure, and policies) is a useful conceptual approach to investigate women’s limited access to cervical cancer screening programs and the impact on health-seeking behaviors. The aim of this paper, which utilized the socio-ecological model as a conceptual framework [12] is to explore and describe micro-, meso-, and macro-structural factors that facilitate or hinder women’s access to cervical cancer screening and prevention services and the implications for cervical cancer prevention among women at risk in Cameroon, a low-income, high HIV prevalence context. The result will highlight current challenges around women’s access to cervical cancer screening in Cameroon and identify potential opportunities in developing and implementing effective interventions for increasing uptake of cervical cancer screening programs.

## Methods

### Ethical approval

The Institutional Review Boards of the Albert Einstein College of Medicine, Bronx, New York and the National Research Ethics Committee in Cameroon granted ethical approval for the study.

### Study setting

The study setting is the Regional Hospital, located in the coastal town of Limbe in Southwest Cameroon. The hospital, built around the 1940s was accorded a provincial status in 1972, thereby becoming the principal referral hospital for the southwest region [13]. Limbe Regional Hospital (LHR) is also called the “Mile One Hospital” because of its location about one mile from the Atlantic Ocean [13]. Since its establishment, the hospital has experienced several structural and organizational problems that have limited its capacity in providing adequate health services for the population it serves.

### Research design and study population

This study utilized an exploratory-descriptive qualitative approach, and was conducted as part of a study assessing cervical HPV infection and neoplastic disease in women living with HIV (WLHIV) that was embedded within the Central Africa International Epidemiology Database to Evaluate AIDS (CA-IeDEA) project in Cameroon [14]. The study population consisted of women living with HIV (WLHIV) and those not living with HIV (HIV-negative) who met the following eligibility criteria: aged >  = 25, ever or currently sexually active, not pregnant at enrollment into the study, never screened for or diagnosed with cervical cancer, was able to provide both self-collected and provider collected biological samples for HPV testing, and was able to understand and sign the informed consent. In addition, male spouses or partners of enrolled women were recruited to explore men’s perspectives and attitudes toward cervical cancer screening.

### Sample selection

We utilized a two-stage purposive sampling strategy to systematically select the women and men who participated in focus group discussions (FGDs) and in-depth interviews (IDIs). The choice of this sampling strategy was informed by the need to ensure different categories of screen eligible women based on HIV status were recruited in the study.

In the first stage, we utilized a master list of 877 women enrolled in the parent study to identify and generate a list of 585 WLHIV and 292 HIV-negative women who met the eligibility criteria. The second stage was a systematic selection of 36 WLHIV with respect to three categories of age: 25–35, 36–45 and >  = 46 years who participated in 3 separate FGD sessions for each age category (12 in each FGD). Additionally, 4 WLHIV were selected among the three categories of age to participate in IDIs. The process was repeated to select the same number of participants among HIV-negative women to participate in FGDs and IDIs. The selection of female participants was done after the study nurses contacted potential respondents, provided information about the study and invited those who were interested to participate in the FGD or IDI to appear at Limbe Regional Hospital for enrollment. Few of the WLWHIV who were approached declined to participate in the study because of time constraints.

In addition, we recruited male spouses of WLHIV and HIV-negative women to participate in separate FGDs. Male spouses were recruited by snowball sampling through asking female participants in each focus group to invite their spouses or significant others to participate in an FGD organized specifically for male spouses. A list of all male spouses who agreed to participate was developed and each spouse was contacted by the study team prior to being invited to participate in the men’s focus group discussions. The first 10 male spouses of WLHIV and the first 10 spouses of HIV-negative women who responded were then invited to join each of the discussion groups.

### Data collection

FGDs and IDIs were held between May and August 2018. The rationale for using FGDs and IDIs was to better understand, and describe women’s knowledge, attitudes, and practices regarding cancers in general, cervical cancer, HPV infection, and cervical cancer screening as well as behavioral and structural facilitators and barriers to cervical cancer prevention. Additional information was obtained to assess and compare perceptions and preferences for self-collected versus health provider-collected biological specimens to explore women’s preferences given peculiar contextual factors, such as stigma that may limit access to cervical cancer screening for women at risk. Focus groups with male spouses and partners explored similar issues, including their knowledge and attitudes about cancer and cervical cancer screening, as well as if and how they provide support to wives and significant others in cervical cancer prevention, treatment and care.

Overall, we organized 6 women and 2 men’s focus groups, and 8 individual in-depth interviews. These consisted of 3 focus groups for WLHIV and 3 focus groups for HIV-negative women in 3 age categories: 25–35 years, 36–45 years and >  = 46 years. Two additional focus groups, (1 for partners of WLHIV and 1 for partners of HIV negative women) were organized for male spouses. Each focus group consisted of 12 people grouped together based on homogeneous characteristics such as age, literacy, socio-economic status and known HIV status category. In addition, 8 in-depth interviews (4 in each category) were held with women study participants. The FGDs and IDIs were held until we achieved saturation in both interview types.

The FGDs and IDIs were held at an unanimously agreed location within the premises of Limbe Regional Hospital that was accessible to only the study participants and Research Assistants (RAs). The venue also afforded anonymity to participants as well as the confidentiality of the information they provided during interviews. The language used during FGDs and IDIs was “*pidgin*”, a colloquial form of the English language widely spoken in the area.

A total of 10 RAs, divided into 2 teams (5 in each team) were recruited to conduct the interviews. Each team, which consisted of 3 females (a moderator, note-taker and observer), facilitated the FGDs and IDIs with WLHIV and HIV-negative women. FGD with male spouses was facilitated by 2 males (a moderator and note-taker). Six [6] of the RAs had a minimum of Masters’ degree in either a social science or public health discipline; the others were studying for a Ph.D. degree in public health. All RAs were experienced in conducting qualitative research and spoke fluent English and pidgin, the local language used to conduct the interviews.

Each FGD lasted 90 min on average while the IDIs lasted an average of 60 min. Each participant in the focus group and in-depth interview received an incentive of approximately $5 (USD) to cover the cost of transportation and other logistics of participation. Prior to starting the group discussion, each participant was provided with a study information leaflet that described the study, its objectives and names of study personnel to contact should the need arise. Thereafter, the RA leading the team discussed the objectives of the study, any potential benefits and/or risks to participants, and reminded participants that participation is entirely voluntary and will not affect their care if they chose to not participate. Participants who agreed to participate were than required to sign the informed consent sheet before the interviews.

### Measures

To facilitate data collection, a semi-structured interview guide that was informed by the socioecological framework [12] was developed by the research team (see Additional file 1: FGD and IDI Guide). The guide was organized around a series of specific themes, each with several open-ended questions. The themes included (i) knowledge, attitudes and behaviors about cervical cancer and association with HPV infection, (ii) individual/interpersonal, socio-economic and cultural influences on cancer screening, including stigma (iii) perceptions of the need for and utilization of cervical and other reproductive cancer screening, (iv) user experiences regarding self-collected versus provider collected methods for biological samples, (v) men’s perceptions, attitudes and support for cervical cancer prevention among women, and (vi) macro-contextual factors, including cost, accessibility and availability of screening services for women in the study areas.

In addition, participants also discussed social-contextual factors that influence beliefs, attitudes, perceptions of personal risk and community actions that can contribute to cancer prevention including integrated models of care HIV and non-communicable diseases such as cervical cancer, role of men in cancer prevention and effective strategies/mediums for disseminating educational materials about cancer prevention. The inclusion of these specific topics in the guide provided valuable insights into the context of cultural and normative factors influencing perceptions, behaviors and the degree to which participants consider access to preventive care as an important component of cervical cancer prevention.

### Data processing

Several steps were taken to process the data generated from the FGDs and IDI. First, at the end of each discussion session and interview, the team of the moderator, note-taker and observer held daily debriefing meetings primarily to reconcile notes from each team member with the audio recording of the FGD or IDI. This step required each team member to listen to the audio recording and cross-check the audio with the notes taken during the interview to ensure consistency and to fill in any gaps in the notes. Upon reconciliation, audio recordings and field notes were labeled with information regarding the location of the interview, basic information about the participant(s), pertinent interview information including, date, starting/ending time of the interview and names of the moderator, note-taker and observer (in case of FGDs). The process contributed to the quality of data generated and ensured consistency in the way the data was managed.

Second, the group that facilitated the discussions and interviews held routine debriefing meetings with the larger research team, which included US based researchers, to give and receive feedback in terms of what they did well and/or needed to improve on for subsequent interviews. Prior to these briefing sessions, other members of the research team who did not participate in the interview listened to the audio recordings so they can provide feedback to the team that facilitated the FGD or IDI. This process helped ensure continuous quality improvement in the data that was obtained as well as in identifying important lines of inquiry needing further exploration during subsequent interviews.

Thirdly, audio recordings were transcribed in “*pidgin*”, the language of communication during the interviews. Translation into English was then done to ensure that those not familiar with *pidgin* were able to understand the information obtained from the FGDs and IDIs. Like the process used in transcribing, translation of transcripts from *pidgin* to English was done by members of the research team who were not part of the group that facilitated the interview/discussion. The transcripts were independently verified, checked for completeness and scanned to ensure all personal identifiers had been deleted.

### Analytical approach

The analytical process began with data immersion, identification of themes and development of a codebook, all of which were handled by the research team. This involved team members doing the following: (i) listened to audio recording of each interview (ii) read field notes taken by interview team, (iii) read original verbatim transcripts and translations to ensure consistency and familiarity with the data prior to identifying themes and developing a codebook. This facilitated the identification/validation of *apriori* themes that were initially developed by the lead author.

The development of the codebook followed a three-stage iterative process. The first stage involved an inductive-deductive analysis of the transcripts, which involved iteratively reviewing, interpreting and discussing verbatim texts of participants’ ideas, opinions, and experiences about cervical cancer. This allowed us to identify substantive themes that emerged from FGDs and IDIs. The second stage entailed a much more detailed analysis of the text, which resulted in identifying and developing codes that were organized based on the themes. The codes that emerged from this process were discussed, and reviewed by the study team, and validated with study participants before they were applied to the transcripts. Disagreements regarding applied codes or whether codes fit into a theme of interest were discussed by the study team to arrive at a consensus. Finally, results were mapped by each domain unto an ecological framework and organized by their relationship to micro, meso, and macro-structural factors regarding cervical cancer in the study setting.

## Results

### Participants’ demographic profile

One hundred participants (80 women and 20 men) contributed data through FGDs and IDIs. Table 1 shows the sociodemographic characteristics of the study participants. Mean age reported was 36.8 years. More than 4 in 10 women were married and more than half reported at least a secondary education. About 41% of women were unemployed and about half (50%) were in small scale self-employment. More than 8 in 10 women reported earning no income or less than 50,000 CFA (USD $83) per month. The mean age at first sexual intercourse was 17.2 years, with most women (46%) reporting a lifetime number of 2–4 sexual partners. One in three women reported current use of an oral contraceptive.

**Table 1:** Demographic characteristics of women in the study

Demographic Characteristics	%
Mean Age	42.6
*Marital Status*	
Single	34.9
Married	44.6
Other	20.5
*Education*	
None	3.2
Primary	11
Secondary	52.8
Tertiary	33
*Employment*	
None	40.6
Self Employed	49.8
Government	9.6
*Income*	
None	42.8
< 50,000 CFA	38.4
> 50,000CFA	18.8
Mean age at sex debut	17.2
*Lifetime sex partners*	
1	8.7
2–4	46
5–6	21
7–9	8.9
10 or more	11.7
Don't Know	3.7
Current oral contraceptive use	33.9

### Major themes and sub-themes

We adopted the socio-ecological model to identify and describe factors that facilitate or hinder access to cervical cancer screening for women in the study community. At the micro (individual) level the major themes that emerged included awareness, knowledge, risk perceptions and behaviors that impact access to and utilization of cervical cancer screening programs. At the meso-level, themes that emerged included familial, social network, community and cultural norms that influence perceptions and utilization of cervical cancer prevention, and the macro-level themes were related to overall macro-structural factors including the health system and policies that facilitate or inhibit access to cervical cancer screening and prevention. These multi-level factors are summarized in Table 2 as potential challenges and opportunities that can impact interventions to improve population-based cervical cancer screening in Cameroon.

**Table 2:** Potential challenges and opportunities in developing and implementing population-based screening in Cameroon according to micro-, meso-, and macro-level factors

	Challenges	Opportunities
*Micro-level (Individual) Factors*		
Awareness and knowledge	Older women were more likely to believe myths and misconceptions about cervical cancer	Nearly all women were aware of at least one type of cancer – cervical and breast cancer most commonly
Risk perceptions and health-seeking behaviors	Limited knowledge of the relationship of HPV and cervical cancer	Younger women were more likely to demonstrate knowledge of risk factors associated with each type of cancer
	Varied perception of risk associated with age, HIV status, adherence to myths and misconceptions and perceived risk of cervical cancer	Nearly all women were aware of increased risk of cancer diagnosis in their community
	All women had never been screened for cervical cancer	Younger women and those with higher education were more likely to take preventive actions to minimize their exposure to risk
		Knowing someone diagnosed with cancer strongly influences perception of risk and willingness to initiative preventative behaviors
		Those that were aware of the risks of cervical cancer were more likely to encourage others to take preventive measures against cervical cancer
Lack of access to information about cervical cancer screening services	Women did not have access to any source to obtain information about cervical cancer which made it possible for false and negative information about cervical cancer to spread in their communities	Women sought information about cervical cancer from internet sources or private health facilities offering screening and other services related to cervical cancer
Cost as a deterrent to cervical cancer screening	Absence of publicly funded cervical cancer screening programs	Available at a few private health facilities, but these services are expensive so many women cannot access them
	Difficulties with personal finances due to high unemployment rates in the country places paying for cervical cancer prevention as low on the list of priorities	Women were likely to appear for cervical cancer screening if it was free and transportation costs were reimbursed
	The cost of transportation to health facilities is an additional financial deterrent	
*Meso-level (Community Norms and Social Networks) Factors*		
Social networks and social norms	The type of information about cervical cancer is determined by the amount of cervical cancer knowledge that community has and how much they are attached to myths and misconceptions about cervical cancer	Community education and stigma reduction around cervical cancer is likely to have a high impact because individual’s knowledge and behaviors are shaped by and conform to expectations is set by the level of awareness in their community
Cultural norms and the role of men	Men do not take much interest in women’s health issues or encourage preventative behaviors as a result of cultural expectations of how men should conduct themselves	Younger women are encouraging men to be proactive in taking concrete action to help prevent their spouses from getting cervical cancer (ie: not having multiple partners, encouraging their wives to participate in regular screening, etc.)
	Men with negative attitudes about cervical cancer believe there is very little to be done to prevent cervical cancer	Men with higher levels of education demonstrated better knowledge of risk factors and was more likely to demonstrate a positive attitude to cervical cancer prevention
HIV and health-related social stigma	Ignorance and fear of death contribute to the stigma surrounding cervical cancer	Lots of opportunity for stigma reduction activities in communities
	The belief that cervical cancer is untreatable fuels stigma	
	Disease associated with women’s reproductive organs contribute to stigma given cultural norms around female sexuality	
*Macro-level (Structural: Health System and Policy) Factors*		
Weak health system and lack of infrastructure	Lack of cervical cancer screening facilities in the regional hospital requires travel to large urban centers for screening	Private clinics have made cervical cancer screening
	Limited basic equipment for screening	
	Shortage of trained health care workers who can keep up with demand	
	Weak health care system and poor condition of physical health centers	
	Emphasis on HIV/AIDS within the health system leaving little space for competing health priorities	
	Shift to private facilities leading to higher costs for patients with limited trust in providers’ skills	
Lack of cancer prevention policies	Lack of comprehensive policies that can aid awareness and encourage positive attitudes to cervical cancer screening	
Cervical cancer screening in in the context of HIV/AIDS care and treatment program	Women not living with HIV or of unknown status did not want to seek screening from services integrated with HIV/AIDS care because of potential HIV-related stigma they may face	Integration of cervical cancer screening within HIV care and treatment programs
		Interest in community-based cervical cancer screening programs which can be accessed in community settings or done in their own homes
Lack of cancer prevention policies	Limited commitment from government and politicians to improve population health	Interest from age-eligible women to be educated on cervical cancer prevention
	Rural–urban disparities in health care infrastructure and supplies	

### Micro level (Individual) factors

#### Awareness and knowledge

Awareness and knowledge of cancers in general and cervical cancer in particular is important in cancer prevention and control. We found that nearly all participants were aware of at least one type of cancer, with cervical and breast cancers being the two most mentioned among female participants. Although most participants mentioned at least one type of cancer, only a few, mostly younger women, were able to demonstrate knowledge of risk factors associated with each type of cancer. Younger women, for instance, reported that sexual intercourse with multiple partners as a lifestyle risk factor in addition to exposure to genetic and/or environmental factors. Older women who subscribed to myths or misconceptions about cervical cancer demonstrated generally poor knowledge of risk factors. Their opinion is reflected in the words of one participant who said:

“…. hmmm vaginal candidiasis, which comes from using unhygienic and unsanitary public restrooms is the reason for many women diagnosed with cervical cancer. In addition, there are lots of women who wear “second hand” clothing, including underwear filled with germs that can cause this problem”. (FGD, Female HIV-negative 36–45 years).

Knowledge of the relationship between HPV and cervical cancer was generally poor. For instance, younger and older participants lacked knowledge that persistent infection with HPV, especially types 16 and 18 is strongly linked to cervical cancer. None of the older women reported having ever heard about HPV, and only a few of the younger women recalled hearing about HPV.

### Risk perceptions and health-seeking behaviors

Risk perception and susceptibility to long term health outcomes are critical to preventive actions to avoid disease acquisition. Most participants acknowledged that a lot of women were at risk of a cancer diagnosis, especially given the increased number of reported cases. With increased cancer incidence, there was a consensus that anyone is susceptible to being diagnosed with cancer if exposed to the risk factors. Perceptions of the severity of risk, however, varied between different categories of participants. For example, older, less educated HIV-negative women were least likely to perceive any risk, whereas older WLHIV were more likely to report higher risk perceptions. Similarly, younger women, regardless of HIV status, reported being vulnerable to some risk factors, for example being in a sexual relationship with in which they or their partners have sex with multiple partners.

Individual perceptions of risk influence preventive behaviors. Although none of the participants had ever been screened for cervical cancer prior to participating in the study, those with higher educational levels or better knowledge of risk factors reported having previously acted or “*did something*” to minimize their exposure to risk. Younger women who discussed environmental, genetic and lifestyle risk factors reported taking preventive actions consistent with their beliefs about their vulnerability. Among older women, there were instances when the reported preventive action was based on myths or misconceptions about cancers. As one respondent reported:

“Like cervical cancer, I’ve heard that allowing antiseptic soap to penetrate the vagina during a bath can cause cervical cancer, therefore, I make sure I do not use antiseptic soap to wash my vagina when taking a bath”. (FGD, Female HIV-negative =  > 46 years).

Despite the consensus that most women were susceptible to cervical cancer, some participants did not consider themselves to be at risk. Those in this category agreed with statements expressed by a participant who said: *“I take care of myself and I am sure I don’t have anything that will bring me cancer”*. We also found that knowing someone who was diagnosed with cancer strongly influences the perception of risk and willingness to initiate preventive behaviors.

Participants who reported family members or friends diagnosed with or having died from cervical cancer were more likely to have higher risk perceptions and to acknowledge the importance of preventive behaviors such as screening and/or obtaining information about cancer prevention. This was particularly common among a group of older WLHIV who reported knowing someone living with or has died from cancer. They were also more likely to encourage others to take active preventive measures or to be better informed about the importance of cervical cancer prevention.

### Lack of access to information about cervical cancer screening services

Access to information about cancers largely influences an individual’s knowledge, risk perception, and health seeking behavior. Our data showed that women in the study communities had limited access to fact-based sources of information about cervical cancer. Few women who sought information on cervical cancer prevention did so by browsing the internet or talking with providers at community-based private health facilities that offered screening and/or services related to cervical cancer. The limited access to information was highlighted by participants as a challenge for those who desired to prevent cervical cancer. Most participants agreed that the absence of reliable, consistent and fact-based information created opportunities for mischievous individuals to spread false, negative and potentially dangerous information about cervical cancer. There was consensus that “*access to reliable and factual sources of information or services about cervical cancer is critical to preventing the high number of deaths that occur due to late diagnosis and presentation”*. One participant in the focus group of younger HIV-negative women indicated that:

“…the limited or complete lack of access to reliable and trusted sources of health information represented a major hurdle for those who know the dangers of cervical cancer, but do not know how to access reliable and trusted information to enable them to take appropriate preventive actions. In an environment rife with myths, misconceptions and mixed messages from the internet, obtaining relevant information about the importance of cervical cancer prevention is critical for women’s preventive behaviors.” (FGD, HIV-negative 25–35 years).

### Cost as a deterrent to cervical cancer screening

In the absence of publicly funded cervical cancer screening programs, women who want to be screened for cervical cancer have limited options, besides the few privately run health facilities, which are often prohibitively expensive and unaffordable for most women. In discussing the cost of services as a challenge to cervical cancer prevention, there was consensus that:

“cost is perhaps the biggest challenge to obtaining cervical cancer screening, not only in the communities but for low-income women everywhere”. Without money, it is impossible to obtain health services even in government run hospitals” (FGD, WLHIV 36–45 years).

In women and men’s FGDs, participants agreed that the economic situation in the country, with high unemployment rates, meant that families struggling to meet their basic needs for daily survival were unlikely to consider paying for cervical cancer prevention given their limited budget,even in situations where they know it can be fatal. Indeed, many women participants reported that they were screening for cervical cancer for the first time only because the service was free (as part of the study). Despite not having to pay for screening, many participants still reported that transportation costs from their homes to the screening center was a major expense that only a few can accommodate within very tight budgets. Most of the women who showed up for screening did so only because they knew they would be reimbursed for their transportation costs. The words of a female respondent captured participants’ feelings about how cost constituted a barrier to accessing screening:

“It would have been impossible for me [and many of us] to show up for the free screening if not that we knew we would be given transport money for coming. I know that if the service was not free, many women will not be able to come, therefore we are grateful to this hospital for this free service and even paying the cost of transportation to come.” (FGD, Female HIV-negative 36–45 years).

Another respondent emphasized this point when she said:

“When they asked me if I want to join the study, I told them I was not interested because I was not sure how much it will cost and I do not have the money to pay for something like this. It was when they told me that I do not have to pay that I agreed to join. How and where would I get the money if I have to pay?” (FGD, Female HIV-negative 25–35 years).

Similar opinions were expressed by male participants who suggested that families that struggled with meeting basic needs for survival will not be able to pay the cost of screening from their meager budget.

### Meso-level (community norms and social networks) factors

#### Social networks and social norms

Social networks and prevailing social norms are critical in shaping individual attitudes and behaviors toward cervical cancer prevention. Our data showed that characteristics of social network members such as age, educational attainment, socioeconomic status, and health-seeking behaviors influenced individuals’ knowledge, attitudes and behaviors. Similarly, the information circulating within an individual’s social network also influenced knowledge or myths or misconceptions that individuals had about cervical cancer.

Women who reported that they knew about cervical cancer risk factors conceded their knowledge is shaped by what they heard, perhaps from the media, but more importantly by the information that is validated or refuted within their social networks. For instance, participants who demonstrated better knowledge of risk factors conceded their knowledge and subsequent behaviors were shaped by expectations set by their social networks. Conversely, women who demonstrated poor knowledge or perception of risk were more likely to have friends and acquaintances who held similar myths or misconceptions about cervical cancer. Thus, social networks and social norms were critical in disseminating and/or validating information about cervical cancer and options for prevention.

### Cultural norms and the role of men

Both male and female participants extensively discussed the powerful influence of cultural norms in shaping men’s attitudes and perceptions towards cervical cancer prevention. Female participants described cultural notions and nuances that often inhibit men from participating in initiatives to promote their own health, much less the health of their spouses. They suggested that men generally do not take much interest in women’s health issues nor encourage preventive behaviors. While there was a consensus that men needed to be proactive in cervical cancer prevention, there were differences between younger and older women in their expectations regarding how much men should be doing to help their spouses prevent cervical cancer. Unlike older women, younger women were more forthcoming in expressing their opinion regarding how men should be more interested in taking concrete actions to help their spouses prevent cervical cancer. As one respondent suggested:

“it should start by not having multiple sexual partners, which increases the risk of passing on a sexually transmitted infection”. (FGD, Female HIV-negative 25–35 years).

Older women shared the view that most men were constrained in terms of what they can do, and that men’s lack of interest in the health of their spouses resulted from cultural expectations and/or notions of how men should conduct themselves. As one participant reported:

“a man who takes too much interest in women’s health runs the risk of being labeled by society and many men want to avoid such perceptions”. (FGD, WLHIV 36–45 years).

Men’s attitudes toward cancer prevention were shaped both by these cultural norms as well as their level of education and knowledge of risk factors for cancer. Generally, male participants with negative attitudes about cancer were those most likely to believe in myths and misconceptions about cancer. Similar to the women who held myths about cancer, some men believed that cervical cancer, for instance, was most likely to be diagnosed among women who took hygiene for granted, used second-hand clothes, had unsanitary toilet habits or were simply promiscuous. Men with such views believed nothing can be done to prevent cervical cancer. A male participant suggested that the way to prevent a diagnosis of cervical cancer is for “*women to avoid second-hand clothing, unsanitary conditions and not sleep around”*. Men with higher levels of education demonstrated better knowledge of the risk factors and positive attitudes for cervical cancer prevention, including actively encouraging spouses to be aware of the risk and getting screened for cervical cancer. One male participant reported that”.

“preventing cervical cancer is a responsibility both men and women should share equally; it should begin with preventing sexually transmitted infections, avoiding risk factors and obtaining screening when possible”. (FGD, Male Partners of HIV-negative Women).

### HIV and health-related social stigma

Disease specific stigma is a barrier to cervical cancer prevention. Ignorance and the fear of death associated with cancers contributed to the pervasive stigma surrounding cervical cancer. The belief that cervical cancer is untreatable was widespread given the number of people reported to have died from one type of cancer or another. This perception continues to drive stigma and, in some cases, the reluctance to screen voluntarily. Often, health conditions with high mortality were generally stigmatized and the perception that cervical cancer was a disease of women’s reproductive organ contributed to stigma given the cultural norms around female sexuality. As some female participants suggested, the association of cervical cancer with either a woman’s reproductive organs or their sexual behavior is generally stigmatized. Some male respondents suggested that “*a woman diagnosed with cervical cancer may have herself to blame*”. One female participant reported that:

“…women’s bodies are subject to all manner of sociocultural regulations and norms that men’s bodies are excluded from. Thus, any condition that affects women, especially their reproductive organ will be stigmatized even if they are not to blame.” (FGD, Female HIV-negative, 25–35 years).

### Macro-level (structural, health system and policy) factors

#### Weak health system and lack of infrastructure

Participants described numerous macro-structural challenges to cervical cancer prevention and control in the study community. Among others, a weak health system, poorly equipped facilities, and poorly trained staff, all of which are necessary for cervical cancer prevention and control were reported by participants as some of the most important structural barriers. Many participants suggested that the lack of cervical cancer screening facilities in the regional hospital means that most women cannot access services within their community unless they go to large urban centers if they desire to be screened. The lack of basic equipment for screening, shortage of trained health workers who can barely keep up with demand, old and dilapidated buildings and lack of comprehensive policies that can aid awareness about and encourage positive attitudes towards cervical cancer screening were identified among the most significant structural barriers.

Additionally, the high prevalence of HIV/AIDS in Cameroon and the focus on increasing access to prevention, care and treatment for people living with HIV/AIDS meant limited resources were available for other health issues including cancer prevention. Only recently has attention began to shift to the importance of addressing HIV associated comorbidities in women at risk. Some participants described the existence of a few private clinics where it is possible to obtain screening for cervical cancer. However, they described additional challenges with accessing services from these private clinics, which included exorbitant cost and lack of trust in providers’ skills. As one participant reported:

“Private clinics are expensive and want to make as much money instead of providing appropriate care. I know of people who started going to a private clinic to receive care but came back to the Mile 1 [the Regional Hospital] because of poor treatment” (FGD Female HIV-negative, 36–45 years).

### Lack of cancer prevention policies

The group of younger more educated women discussed challenges at the policy level. First, they highlighted the extensive focus on HIV/AIDS, which has resulted in a lack of attention to other important health issues, such as cervical cancer and suggested that more needs to be done regarding cervical cancer prevention. They also cited the ongoing political situation and marginalization, which meant that government officials only pay lip service to improving population health. Further, they discussed the rural–urban disparity in which health facilities in larger urban areas were better equipped and staffed compared to semi-urban or rural ones.

### Cervical cancer screening in the context of HIV/AIDS care and treatment program

The integration of cervical cancer screening within general HIV care and treatment programs has been recommended as an effective way to increase access to screening for women at risk. We assessed participants’ perceptions and attitudes to this approach in increasing access to cervical cancer screening. The WLHIV already in care agreed that such an approach was beneficial as they were able to continue their treatment for HIV and at the same time be screened for cervical cancer. This helped them reduce their risk, number of hospital visits, cost implications and potential stigma if the services were separated. Women of unknown HIV status or those who previously tested negative for HIV, however, objected to the idea of an integrated approach. The stigma of HIV and the fear of being seen going into an HIV treatment center and/or suspected of living with HIV was cited as an impediment to their ability to utilize cervical cancer screening offered within HIV care and treatment programs.

Similar opinions were expressed by male participants who suggested that it would be difficult for women, especially if they were reluctant to test for HIV and/or do not want to be seen going into an HIV clinic because of stigma. They indicated that it was not a good idea to combine cervical cancer screening with HIV testing only because women who go in for cervical cancer screening may be suspected of having HIV even if they do not. Both female and male participants, however, expressed positive attitudes and support for community-based cervical cancer screening programs in which women had the opportunity to access screening within their homes or in community settings.

## Discussion

Invasive cervical cancer is a leading cause of death among women in low and middle-income settings. Although cervical cancer is largely preventable, women in LMICs are disproportionately burdened because they lack access to prevention and treatment strategies that are widely available in high-income settings. Increasingly, cervical cancer prevention programs, including population-based HPV vaccination, are now being implemented in several countries in sub-Saharan Africa. These programs are beset by challenges and are yet to have the desired impact in reducing morbidity and mortality due to invasive cervical cancer [4, 15, 16]. The failure of existing programs is partly due to a lack of understanding of multi-level socio-contextual determinants of access, utilization, and effectiveness of existing interventions, which are important to reduce incidence and mortality and eliminate disparity in access to care. Our study explored these multi-level socio-behavioral and contextual-structural factors that are vital to the success of cervical cancer prevention programs in sub-Saharan Africa.

In this study of the challenges and opportunities associated with cervical cancer screening program for women in low-income, high HIV prevalence in Limbe, Cameroon, we found major themes related to micro-, meso- and macro-level factors, which facilitated or hindered access to and utilization of cervical cancer prevention programs. At the micro-level, women’s knowledge about cervical cancer critically determined attitudes and behaviors towards cervical cancer prevention. Similar to findings in other studies [17–19], women in Limbe were aware of the grave risk that cervical cancer poses for health. They demonstrated high-risk perception by indicating that anyone was at risk given the number of people they know who were diagnosed with or had died from cancer. Regardless, they had poor knowledge of risk factors including sexual transmission of HPV as the main risk factor in cervical cancer. This finding supports the conclusion from previous studies [20] on the need to develop and implement theory-driven population-based cervical cancer educational programs in this study setting and similar ones across sub-Saharan Africa.

Poor knowledge of risk factors coupled with the high mortality from late diagnosis, contributes to a widespread belief that cervical cancer is incurable and ultimately leads to death. In many settings, people with diseases that are deemed incurable are often stigmatized because of their implications for health and wellbeing [21–23]. The perception of cervical cancer as an incurable disease makes it important to target stigma reduction as a component of any educational intervention aimed at improving knowledge and attitudes and encouraging positive preventive behaviors. The stigma reduction framework proposed by Stangl and colleagues [24] could be a useful starting point to eliminate the stigma of cervical cancer.

At the meso level, family, social network and community characteristics powerfully shape women’s knowledge and behaviors about health, including cervical cancer, as well as access to resources for support. The importance of social networks in influencing health-related knowledge and behaviors have been previously described [25–27]. Studies have also documented ways in which social networks can be important sources of support for promoting health and wellbeing [28–30]. Individually and collectively, members of social networks provide social, emotional and financial support for those dealing with a health issue. In this study, we found that characteristics of social network members, including age, education, knowledge, attitudes, and perceptions about cervical cancer, in turn, shaped women’s opinions and preventive behaviors. Given the important role of social networks, it is necessary to consider interventions that empower social networks so they can promote positive behaviors among their members.

Human papillomavirus is the viral agent in invasive cervical cancer. The heterosexual transmission of HPV especially types 16 and 18, which have been linked to invasive cervical cancer, highlight the important role of men in cervical cancer prevention. Male responsibility should focus on reducing the risk of HPV transmission, for example by limiting the number of sexual partners or using condoms and enhancing their roles as cultural gatekeepers/heads of households to encourage women and girls to receive the HPV vaccination.

Similar to other studies that have explored male involvement in cervical cancer prevention, we found that most male respondents had poor knowledge of HPV as a risk factor in cervical cancer [31–33]. Consequently, there is an urgent need to implement educational interventions aimed at increasing men’s knowledge of risk factors and empowering them to be more proactive in preventing cervical cancer. Furthermore, the cultural norms of masculinity that dictate roles and set expectations that discourage male involvement in promoting women’s reproductive health or their ability to engage in cervical cancer prevention should be addressed.

Inadequate health infrastructure and lack of resources for cervical cancer prevention in many settings in SSA has informed strategies to integrate cervical cancer screening within HIV care and treatment programs. A systematic review by Sigfrid, et. al., [34] suggested that interventions based on different models of service delivery may be feasible and acceptable to women. However, our findings suggest that this may not necessarily apply in our study setting and similar ones. The stigma of HIV still presents as a major challenge for women willing to undergo screening for cervical cancer in contexts where such screening is offered within HIV/AIDS prevention, care, and treatment programs. Persistent HIV stigma will limit women’s access to cervical cancer screening programs that are integrated within HIV treatment programs. This makes it unlikely that the WHO’s recommendation of integrating cervical cancer prevention within HIV programs will have the desired impact in communities where HIV stigma is intense. Therefore, for this approach to succeed in increasing access, intervention programs must consider the cultural nuances and community attitudes around health-related stigma so as to better understand how this can affect women’s preferences for accessing cervical cancer screening that is integrated within HIV programs.

At the macro-structural level, a weak health system, poorly equipped health facilities, shortage of trained personnel, out of pocket cost to access screening and the absence of population-based cervical cancer prevention programs continue to inhibit women’s abilities in preventing cervical cancer. Without government led population-based program and policy initiatives aimed at reducing the incidence of cervical cancer and improving access to care and treatment for women at risk, it is likely that Cameroon, a high HIV burden country, will also remain a high burden country for cervical cancer. Presently, non-governmental actors that depend on external funding sources dominate the landscape in providing access to prevention and treatment for women at risk. Based on lessons in other settings [35, 36], implementing structural reforms to ensure women have access to appropriate, cost-effective and user-friendly options while strengthening health system capacity for cervical cancer prevention is critical in addition to continuing to empower non-governmental actors in providing care to women most at risk.

## Strengths and limitations of the study

Knowledge of the socio-contextual barriers to women’s access cervical cancer screening and care in a high HIV prevalence, low-income contexts is a strength of our study. Our findings highlight challenges that program managers and policy makers need to consider in strategies to increase access to cervical cancer screening. Despite the strength, we caution that the interpretation of the results should be considered in light of the following limitations: the qualitative nature of the data, the participants selected and more importantly, the setting of the study within the context of a larger ongoing clinical study- all of which may impact the extent to which results are generalizable to women in dissimilar settings.

## Conclusion

The findings presented in this paper demonstrate the importance of the individual, familial, community and structural factors that can facilitate or hinder access to cervical cancer screening for women at risk in the study community. These issues should be considered when implementing strategies to increase access to cervical cancer screening for women in Cameroon and low-income similar contexts. More specifically, our results highlight gaps in the uptake of cervical cancer screening programs, which must be addressed to increase uptake in cervical cancer screening among women in Cameroon.

## Supplementary Information

**Additional file 1.** FGD and IDI Guide.

## Data Availability

The datasets used and/or analyzed during the current study are available from the corresponding author on reasonable request.
