# Supplementary material for: Challenges and opportunities associated with cervical cancer screening programs in a low income, high HIV prevalence context
Source: BMC Womens Health. 2021 Feb 18;21:74. doi: 10.1186/s12905-021-01211-w (PMC7890622; doi:10.1186/s12905-021-01211-w)
Supplement: Supplementary file 1 — Additional file 1. FGD and IDI Guide. [file 12905_2021_1211_MOESM1_ESM.pdf]

Focus Group and Individual Interview Guide for women undergoing cervical cancer screening in Limbe, Southwest Cameroon

Introduction

Welcome and thank you for joining us. My name is (**introduce facilitator**). I am working with investigators from the Albert Einstein College of Medicine in New York and Clinical Research Education, Networking and Consultancy (CRENC) in Yaounde, Cameroon, who are conducting a cervical cancer-screening study in Limbe Regional Hospital. With me today is (**introduce note taker**) who will be helping me to take notes of important issues during our conversation. We have requested you to join us today so we can learn from your experiences and hear your perspectives on a range of issues about the cervical cancer-screening study in which you participated. We expect to be together for a total of 60-90 minutes.

Today, the focus of our conversation will be on 4 key areas, which include the following:

1. We will talk about your knowledge, attitudes and behaviors regarding cervical cancer and associations with HPV infection
2. We will discuss personal and structural facilitators and barriers to screening for cervical and/or breast cancer
3. We will talk about your perceptions and preferences for self-collection versus provided collection options for screening
4. We will talk about knowledge, attitudes and behaviors of people in this community to combined HIV and NCD screening, like screening for cervical cancer, versus HIV testing alone.

Please note that participating in this discussion/interview is entirely voluntary. You may choose to leave now or any time during the interview. You should be aware that you are not under any compulsion to respond to any of the issues we ask you if you are not comfortable. Your decision will not affect your rights in anyway. We have made arrangements to reimburse you for the cost of your travels to participate in this meeting. Please feel free to ask any question now or after the discussion. Before we proceed, we will need you to complete the informed consent sheet, which will indicate that you are here of your own free will and voluntarily consent to share your opinions, experiences and perceptions with us.

I also want to inform you that we will be audio recording our conversation on a voice recorder so that we will not forget all the important information you will share with us today. I hope you do not mind having our conversation audio recorded, but if you have any objections to being recorded, please let me know and we will switch off the recorder.

We will now begin the discussion.

Please introduce yourselves. You may choose to use your first name(s) or a pseudonym, which we will use to address you during this meeting

Exploring women's knowledge, attitudes and behaviors regarding cervical cancer, other reproductive cancers and associations with HPV infection

Can you tell me the common diseases that are unique to women and their reproductive health?

What factors are responsible for these diseases? [Probe- biology, environment, culture, lifestyle and behaviors, role of men, others]

What category of women is mostly at risk for these diseases? [Probe- age, SES status, education, marital status, others?] Why are these specific women most likely to be impacted?

What do you know about cancers? Describe cancers that are common among women in this town [Probe- specific cancers unique to women, e.g. cervical, breast, ovarian, etc.]

What do you think cause these cancers? [Probe- different causal factors/reasons for specific cancers mentioned, knowledge of HPV and cervical cancer]

How can we prevent cancers? [Probe- education, lifestyle, screening community action, etc.] How would these preventive measures vary by different types of cancers?

What have you done to prevent cancers? What informed these actions/behaviors? Does the same apply to other women within and outside your social networks

What do you know about screening for cervical cancer? Do you think women understand the importance of cervical cancer screening?

Explore individual and structural facilitators and barriers to screening for cervical and/or other reproductive cancers

Describe the actions/steps you have taken or currently taking to prevent onset of cancers [Probe- specific actions regarding cervical cancers]

What factors motivate or encourage you to take these actions/steps [Probe- Micro- individual, Meso- family/community, Structural- availability, access and affordability of screening/treatment services]

What factors hinder or demotivate you from taking these actions/steps [Probe as above]

Describe what you have done to overcome these barriers [Probe- how is this similar or different from what other women do?]

What can be done to empower women to prevent getting diagnosed with reproductive cancers? [Probe- role of specific actors at family, community and societal levels]

What roles should the following groups play in preventing cancers both in themselves and their spouses or loved ones? [Probe- Wives, Husbands, cultural gatekeepers, government, etc.]

Assess and compare women's perceptions and preferences for self collected versus provided collected options for biological specimens

Describe your experiences when you were screened for cervical cancer in Limbe Hospital [Probe- method of recruitment, consent process, attitude of health workers, screening environment, quality of information provided, follow up activities, sharing of results, confidentiality, etc.]

With which of the above factors were you most impressed? With which were you most unhappy? What could we have done differently to enhance your experience of the screening process?

What are your thoughts/experiences regarding the self-collected versus provider collected specimen? [Probe- which do you prefer and why? Which do you think most women would prefer and why?]

Given your experience, what are your thoughts between hospital-based versus home/community-based cervical cancer screening? [Probe- what challenges do you anticipate for each option?]

How do you think most women will choose when given the option between (i) hospital-based versus home/community-based screening? (ii) self-collected versus provider-collected specimen? [Probe specific reasons for responses/opinions)

Assess knowledge, attitudes and behaviors related to combined HIV and NCD testing versus HIV testing alone

How would you describe the attitude of people in this community to HIV testing? [Probe for positive or negative attitudes and why?]

What factors inhibit people from testing for HIV in this community? [Probe- how are these similar or different for men, women, adolescents, key populations?]

What should be done to increase HIV testing among people in this community? [Probe- how similar or different should strategies to increase HIV testing be for different groups- men, women, adolescents, key populations]

What are your thoughts on being offered HIV testing along with screening for cervical cancer and other non-communicable diseases? [Probe-Why should screening for other diseases or conditions be combined with HIV testing versus testing for HIV by itself?]

In what ways do you think this will help overcome the barriers to increasing uptake in HIV testing? [Probe if this is similar or different for men, women, adolescents, key populations]

Closing

Thank you so very much for your time as well as the thoughts, experiences, perceptions and opinions you shared with us. We have come to the end and we want to express our gratitude for the time you have spent with us. We will be available to answer your questions if you have any. Have a safe trip home.
